# Neurocognitive Performance and Executive Functions Do Not Influence Conditioned Pain Modulation in Women with Migraine

**DOI:** 10.3390/life16010027

**Published:** 2025-12-24

**Authors:** Juan C. Pacho-Hernández, Angela Tejera-Alonso, Ana I. de-la-Llave-Rincón, Silvia Ambite-Quesada, Cristina Gómez-Calero, Ricardo Ortega-Santiago, César Fernández-de-las-Peñas, Gustavo Plaza-Manzano, Juan A. Valera-Calero, Margarita Cigarán-Méndez

**Affiliations:** 1 Department of Research and Psychology in Education, Universidad Complutense de Madrid, 28040 Madrid, Spain; jpacho@ucm.es; 2 Department of Psychology, Universidad Rey Juan Carlos, 28922 Madrid, Spainmargarita.cigaran@urjc.es (M.C.-M.); 3 Department of Physical Therapy, Occupational Therapy, Physical Medicine and Rehabilitation, Universidad Rey Juan Carlos, 28922 Madrid, Spain; anaisabel.delallave@urjc.es (A.I.d.-l.-L.-R.); silvia.ambite.quesada@urjc.es (S.A.-Q.); cristina.gomez@urjc.es (C.G.-C.); ricardo.ortega@urjc.es (R.O.-S.); 4 Department of Radiology, Rehabilitation and Physiotherapy, Universidad Complutense de Madrid, 28040 Madrid, Spain; gusplaza@ucm.es (G.P.-M.); juavaler@ucm.es (J.A.V.-C.); 5 Grupo InPhysio, Instituto de Investigación Sanitaria del Hospital Clínico San Carlos (IdISSC), 28040 Madrid, Spain

**Keywords:** migraine, conditioned pain modulation, attention, memory, processing

## Abstract

**Introduction**: Migraine is featured by altered nociceptive processing and the presence of cognitive impairments. No study has previously investigated the influence of neurocognitive performance and executive functions in descending pain processing in this population. **Aim**: To assess the influence of neurocognitive processes and executive functions in conditioned pain modulation (CPM) activation in women with migraine. **Methods**: A cross-sectional case–control study including 140 women with migraine (50% chronic) and 70 control women was conducted. Clinical migraine features, neurocognitive processes (e.g., attention), and executive functions (memory, mental inhibition, speed of processing) were evaluated. Pressure pain thresholds (PPTs) were bilaterally assessed at the temporalis muscle, lateral epicondyle, and tibialis anterior muscle. Heat (HPT) and cold (CPT) pain thresholds were assessed at the frontalis (trigeminal area) muscle. Thus, CPM was evaluated with the cold pressor test paradigm by analyzing changes in mechanical/thermal stimuli after a conditioned stimulus. **Results**: Significant group*time interactions not associated with neurocognitive process/executive function, educational level, and employment status were found for PPTs at the temporalis muscle (Wilk’s λ = 0.588, F[2,199] = 69.756, *p* < 0.001, n^2^p = 0.412, 1 − β = 0.999), lateral epicondyle (Wilk’s λ = 0.674, F[2,200] = 48.331, *p* < 0.001, n^2^p = 0.326, 1 − β = 0.999), and tibialis anterior (Wilk’s λ = 0.751, F[2,200] = 33.110, *p* < 0.001, n^2^p= 0.249, 1 − β = 0.999): PPTs were higher after the conditioned stimulus in all points in control women (increases ranging from 11% to 17%), whereas PPTs were lower after the conditioned stimulus in women with migraine (decrease from −7.5% to −0.1%) when compared with PPTs at baseline. Changes in HPT and CPT were small and not significant, ranging from 0.1% to 0.5%. **Conclusion**: This study revealed that women with episodic or chronic migraine showed CPM deficits particularly against mechanical stimuli when compared with pain-free women. Neurocognitive (e.g., attention) processes or executive functions (e.g., working memory, mental inhibition) did not modulate CPM activity in women with migraine.

## 1. Introduction

Among primary headaches, migraine is a disabling pain condition showing a one-year prevalence of 15% in the general adult population [1]. In fact, migraine is the leading cause of disability in people under the age of 50 years, particularly in women [2]. Since its highest prevalence occurs during the most active working years (30–40 years), its impact extends to social functioning, professional productivity, and economic costs [3].

Current theories contemplate migraines as a multifactorial condition where several mechanisms may interact simultaneously [4]. One conceptual model claims the presence of altered nociceptive gain processing, i.e., peripheral and central sensitization mechanisms, as a key element in the transition from episodic toward chronic migraine [5]. Thus, an altered pain processing may manifest as a facilitated state, that is, an exaggerated response (e.g., hyperalgesia or allodynia) to different stimuli (e.g., thermal, mechanical, or electrical) [6,7] and/or as an inhibitory state, reflected as deficits in descending inhibitory pathways (i.e., impaired conditioned pain modulation) [8]. Conditioned pain modulation (CPM) reflects the activation (or lack of) of descending inhibitory pain mechanisms where a painful stimulus in one part of the body inhibits pain in another [8]. Deficits in CPM have been associated with an impaired pain inhibition, which means a facilitatory pain state [8]. Evidence on CPM deficits in individuals with migraines is inconsistent. Some studies have identified impaired CPM in migraine sufferers when compared with controls, while others did not [9,10]. Authors of both reviews concluded that discrepancies may be attributed to factors such as the following: 1, large variability in CPM paradigms used (i.e., electrical, mechanical, or thermal); 2, small and mixed-gender samples; 3, lack of distinction between episodic or chronic form; and/or 4, lack of control for cognitive influences on pain processing [9,10].

Cognitive alterations are self-reported by up to 80% of patients during a migraine attack; however, this prevalence is dramatically decreased when objective measures are used [11]. In fact, cognitive alterations in patients with migraine range from concentration problems, memory problems, and decreased mental processing speed [12]. Although these symptoms can be present during and between headache attacks, deficits in cognitive performance, particularly selective attention and executive functions, are most affected during migraine attacks [13]. The presence of cognitive alterations in individuals with migraine is explained by the cortical spreading depression, a phenomenon occurring during migraine attack that increases sensitivity to external stimuli while compromising focus, decision-making, and attentional control [14]. Nevertheless, no previous study has investigated the influence of neurocognitive performance and executive functions in descending pain processing in migraine.

Accordingly, we conducted a case–control study aimed to evaluate endogenous pain modulation in women with migraine by including neurocognitive processes and executive functions. Therefore, the aim of the current study was to evaluate the influence of neurocognitive performance and executive functions in CPM activity in women with migraine, attending to their chronicity.

## 2. Methods

### 2.1. Participants

A case–control study including women with episodic or chronic migraine and pain-free healthy women following the Strengthening the Reporting of Observational studies in Epidemiology (STROBE) guidelines [15] was conducted. Consecutive women who visited the Headache Unit at the Neurology Department of Hospital Universitario Fundación Alcorcón (HUFA) from March to September 2025, an urban hospital in Madrid (Spain), with headache were eligible to participate. To be included, they must be diagnosed with migraine according to the third edition of the International Headache Society (IHS) [16] by an experienced neurologist. The location and quality of pain, years with migraine, family history, and medication intake were collected from medical records. Additionally, participants were asked to complete a 4-week headache diary documenting the following: (1) number of migraine days per month; (2) duration of migraine attacks (hours/episode); and (3) migraine intensity measured by the Numerical Pain Rating Scale (NPRS, 0–10 points) [17].

Participants were excluded if they had the following: 1, coexistence of other primary/secondary headache [16]; 2, previous neck trauma (i.e., whiplash) or cervical disk herniation; 3, any comorbid medical disease altering pain processing (e.g., fibromyalgia syndrome); 4, any psychiatric diagnosis (e.g., major or mild neurocognitive disorders, schizophrenia) based on the DSM-V; 5, intake of any drug with potential cognitive effects (e.g., antipsychotics, anticonvulsants, or anticholinergics); 6, pregnancy; or, 7, receipt of any type of treatment, including anesthetic blocks, in the 3 months prior to enrollment.

For the control group, we recruited women without a history of migraine or other recurrent headache and who had not experienced any headache episode at least during the previous year. Controls were matched for age with the migraine group and underwent neurological examination to confirm eligibility.

The study design has been approved by the Local Ethics Committees of the institutions involved (HUFA 24_117; URJC_010220240912024). All participants were informed of the study and signed the informed consent before their participation. All procedures were performed following the ethical standards of the Declaration of Helsinki.

### 2.2. Neurocognitive Processes

**Figure 1:**
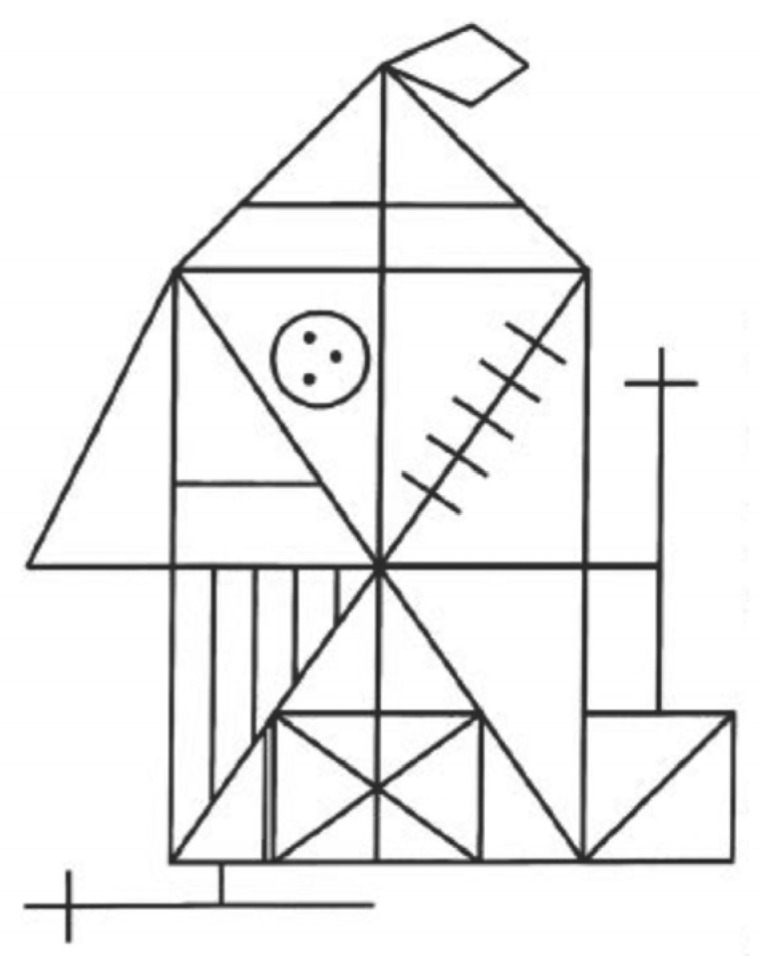
The Rey–Osterrieth Complex Figure (ROCF) [18].

Visual perception, visual construction ability, and incidental memory retention were examined using the Rey–Osterrieth Complex Figure (ROCF) [18]. In this task, subjects are instructed to reproduce a complex geometric figure composed of 18 black lines (Figure 1). The drawing is first copied directly, then it is reproduced from memory both immediately (immediate recall) and again after a 20–30 min delay (delayed recall). Since no request to memorize or instructions are given, the procedure evaluates spontaneous retention. Three scores are obtained: ROCF_Copy (direct score during the copy phase), ROCF_Recall (percentage recall calculated as delayed recall divided by immediate recall), and ROCF_TimeCopy Copy (time needed to complete the copy).

**Figure 2:**
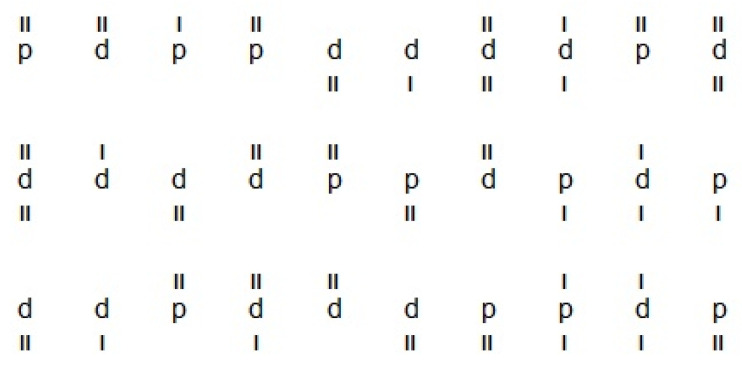
Example of d2 Attention Test (d2) [19,20].

Selective attention and concentration were assessed with the Spanish adaptation of the d2 Attention Test (d2) [19,20]. This instrument presents 14 rows of 47 characters each (658 stimuli in total). Participants must rapidly scan each line and mark every “d” carrying exactly two small dashes (positioned above, below, or one above and one below). These “d” letters constitute the target stimuli, while distractors include “p” or “d” with fewer or more than two dashes (Figure 2). Each row is performed under a 20 s time limit, with the full test lasting approximately 8–10 min. Derived indices are d2_TR (total attempted items), d2_TA (correctly identified targets), d2_O (number of missed targets), d2_C (errors by marking irrelevant stimuli), d2_TOT (overall performance efficiency calculated TR-(O+C)), d2_CON (concentration index, calculated TA-C), d2_TR+ (row with maximum attempts identified), d2_TR− (row with minimum attempts), and d2_VAR (variation index, calculated TR+ minus TR−).

### 2.3. Executive Functions

**Figure 3:**
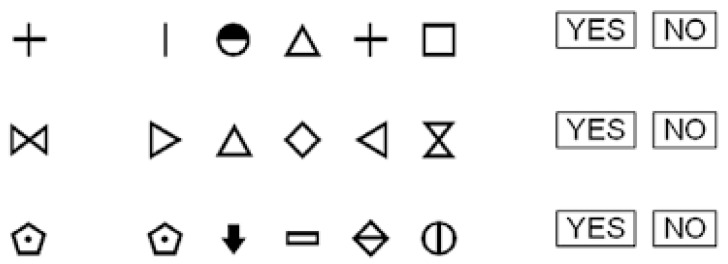
Example of Symbol Search (SS) subtest from the WAIS-IV [21].

Processing speed was measured with the Symbol Search (SS) subtest from the Wechsler Adult Intelligence Scale (WAIS-IV) [21]. In this task, a key panel displays nine meaningless pairs of numbers and symbols, while a response panel presents the numbers randomly placed next to empty boxes (Figure 3). The participant’s task is to insert the corresponding symbols in the blanks as quickly as possible within a 120 s limit.

**Figure 4:**
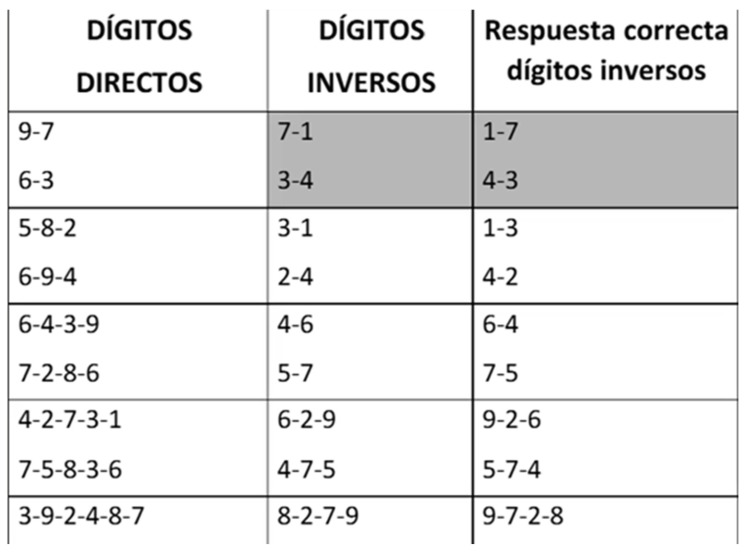
Example of Digits D/R/I subtest of the WAIS-IV [22].

Working memory was evaluated with the “Digits D/R/I” subtest of the WAIS-IV (Figure 4) [22]. It includes three subtasks: 1, digit span forward (DSF), in which participants repeat orally presented sequences of numbers in the same order; 2, digit span backward (DSB), requiring reproduction of the sequence in the reverse order; and 3, digit span sequencing (DSS), where numbers are reordered and repeated in ascending order.

Mental inhibition/inhibitory control was assessed with the “response inhibition index” of the 5-Digit Test (FDT) [23], a Stroop-like paradigm with four sections of 50-items each: Reading, Counting, Election, Alternation. Reading and Counting capture more automatic and basic processes, whereas Election and Alternation require higher cognitive control. Performance is quantified by multiplying the number of errors by the completion time for each part. The following scores are given: Decoding_FDT (time needed to read numeric items), Retrieving_FDT (time needed to process non-numeric items such as the asterisks), Inhibiting_FDT (time needed to repeat the same numeric item), and Shifting_FDT (time needed to process mixed numeric items within boxes).

**Figure 5:**
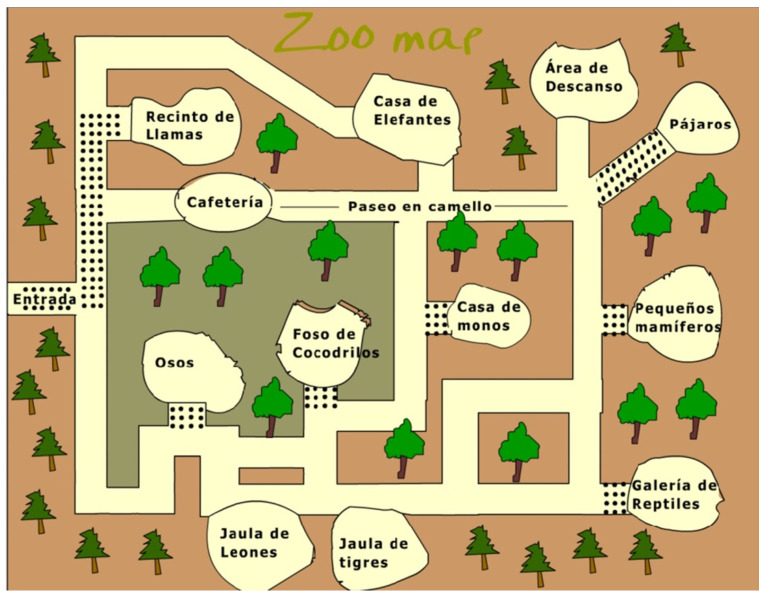
The Zoo Map Test [24].

Planning and decision-making were tested with the Zoo Map Test (Figure 5) [24], which consists of two parts: the first one evaluates planning ability in an unstructured context without predefined rules, while the second one assesses the ability to apply an external structured strategy. In both parts, errors are subtracted from the sequence score, and the final total score (0–16) is obtained by summing both parts.

### 2.4. Psychophysical Outcomes: Pressure and Thermal Pain Thresholds

Pressure pain thresholds (PPTs) were assessed bilaterally at three different anatomical locations: the temporalis muscle (trigeminal site), the lateral epicondyle (extra-trigeminal site), and the tibialis anterior muscle (remote site) with an electronic algometer (Somedic AB, Farsta, Sweden). The pressure was applied approximately at a rate of 30 kPa/s. For each location, three randomized trials were performed with a 30 s pause between trials to minimize temporal summation. The mean of the three repetitions was calculated and used for statistical analysis.

Pain thresholds to heat (HPT) and cold (CPT) were bilaterally calculated with a thermal stimulator (ATS System, Medoc Pathways System, Israel) on the frontalis muscle (trigeminal area innervated by V1 branch). Three repetitions were again obtained with an interval of 30 s, and their mean values were used for statistical analysis.

### 2.5. Conditioned Pain Modulation Paradigm

An evaluator blinded to the women’s conditions conducted all procedures. For women with episodic migraine, testing was conducted on a migraine-free day and only if at least seven days had elapsed since a previous migraine attack, thereby minimizing the risk of migraine-related allodynia. For participants with chronic migraine, assessments were conducted on a migraine-free day when possible, and to ensure consistency, at least 1–2 days after the most recent migraine attack. If not possible, evaluation was otherwise conducted when pain intensity was rated <3/10. Participants were instructed to abstain from analgesics or muscle relaxants for a minimum of 48 h before testing. Prophylactic treatments were left unchanged.

The CPM paradigm used in the current study was the cold pressor test [25] which has exhibited good to excellent reliability [26]. First, PPTs, HPTs, and CPTs were calculated as previously described. Second, participants immersed one hand (the hand of the side of migraine in women with unilateral migraine or the dominant hand in women with bilateral migraine or control women) for 1 min in a water container (Huber K20-cc NR, Offenburg, Germany) at a temperature of 10 °C. Pain intensity during immersion was not collected, as focusing on the conditioning stimulus is known to diminish CPM response [27]. Third, participants removed their hands from the water, and PPTs, HPTs and CPTs were immediately assessed again following the same procedure described above.

An impaired CPM is operationally defined as no change or even a negative change in data obtained before and after the conditioned stimulus [28]. Two scores were computed: (1) the absolute change (kPa or °C; CPM change score) and (2) the relative percentage difference (%, CPM activation index) between pre- and post-conditioning values.

### 2.6. Sample Size Calculation

The “a priori” estimated sample size was calculated using the G*Power 3.1.9.7 computer program. Statistical parameters were set for an F test with repeated measures focusing on the primary study outcome, CPM. The statistical inputs were effect size (f^2^): 0.25, significance level (α): 0.05, statistical power: 0.90, three groups, and two number of measurements. Based on these criteria, the analysis indicated that a minimum of 206 participants would be required.

### 2.7. Statistical Analysis

The SPSS Statistical Software (version 27.0) was used for statistical analyses. Prior to testing, assumptions of normality and sphericity were verified. Continuous variables are reported as means (standard deviations), while categorical variables are expressed as frequencies (percentages). Intergroup comparisons (episodic migraine, chronic migraine, controls) were first explored using one-way ANOVAs for continuous data and chi-square tests for categorical variables to identify covariates for subsequent analyses.

The primary analysis included separate multivariate repeated-measures analysis of covariance (OMNIBUS RM-ANOVA) to identify the effect of migraine on CPM (change in PPT, HPT, CPT). Demographic variables, neurocognitive processes, and executive functions differing among groups were entered as covariates (cofounder covariables). In this model, mechanical and thermal pain thresholds across time (before/after conditioned stimulus) were the within-subject factor, group (episodic migraine, chronic migraine, controls) was the between-subjects factor, and all variables significantly different among the groups were included as covariates. We assessed multicollinearity among the neurocognitive variates included in the RM-MANCOVA using Variance Inflation Factor (VIF), Tolerance indices, and Pearson correlation coefficients. All VIF and Tolerance values were within acceptable ranges (VIF below the problematic threshold of 5 and Tolerance above 0.20), and all Pearson correlation coefficients were below 0.80, indicating no multicollinearity among the neurocognitive variables/executive functions. Residual normality was examined through the Kolmogorov–Smirnov test and visual inspection of Q–Q plots, showing that residuals were normally distributed. The neurocognitive variables included in the MANCOVA were selected because in prior analyses among the groups, they were the variables that showed statistically significant between-group differences and represent theoretically relevant cognitive domains likely to influence the study outcomes.

Adjustment for multiple comparisons was performed, and statistical significance was set at *p* < 0.015 (Bonferroni correction: 0.05/3). Effect sizes were calculated with partial eta squared (η^2^p), interpreted as small (0.01), medium (0.06), or large (>0.14) [29]. Bonferroni post hoc tests were conducted to specify intergroup differences.

## 3. Results

### 3.1. Descriptive Data and Intergroup Comparisons

Sociodemographic and clinical data of the sample are depicted in Table 1. Chi-square analyses showed significant intergroup differences in educational level (*p* = 0.006) and employment status (*p* < 0.001).

Table 2 shows between-group comparisons of neurocognitive performance and executive function. One-way ANOVA revealed significant intergroup differences in DSS (*p* = 0.034), ROCF _Recall (*p* = 0.020), ROCF_TimeCopy (*p* = 0.028), Decoding_FDT (*p* = 0.001), Retrieving_ FDT (*p* = 0.001), and Zoo Map test (*p* = 0.003): women with episodic migraine showed higher scores in DSS (mean difference: 0.9 points, 95%CI 0.1 to 1.8, *p* = 0.030) and lower scores in Decoding_FDT (mean difference: −1.8 points, 95%CI −3.5 to −0.2, *p* = 0.021) and Retrieving_FDT (mean difference: −3.2 points, 95%CI −5.8 to −0.8, *p* = 0.006) than women with chronic migraine. In addition, control women had higher scores in ROCF_Recall (mean difference: 2.9 points, 95%CI 0.4 to 5.4 *p* = 0.018) and lower scores in ROCF_TimeCopy (mean difference: −0.5 points, 95%CI −0.9 to −0.05, *p* = 0.023), Decoding_FDT (mean difference: −2.6 points, 95%CI −4.3 to −0.9, *p* = 0.001), Retrieving_FDT (mean difference: −3.7 points, 95%CI −6.2 to −1.2, *p*= 0.001), and Zoo Map test (mean difference: −1.8 points, 95%CI −3.2 to −0.6, *p* = 0.002) as compared to women with chronic migraine.

### 3.2. Mechanical CPM in the Trigeminal Area (PPTs Temporalis Muscle)

The repeated-measures MANCOVA (the estimated marginal means and standard deviations can be seen in Table 3) exhibited a significant time effect for PPTs (Wilk’s λ = 0.862, F[1,199] = 31.905, *p* < 0.001, n^2^p = 0.138, 1 − β = 0.999): PPT values were higher after the conditioned stimulus (mean difference: 14.0 kPa, 95%CI 9.15 to 18.9, *p* < 0.001) than before the stimulus.

**Figure 6:**
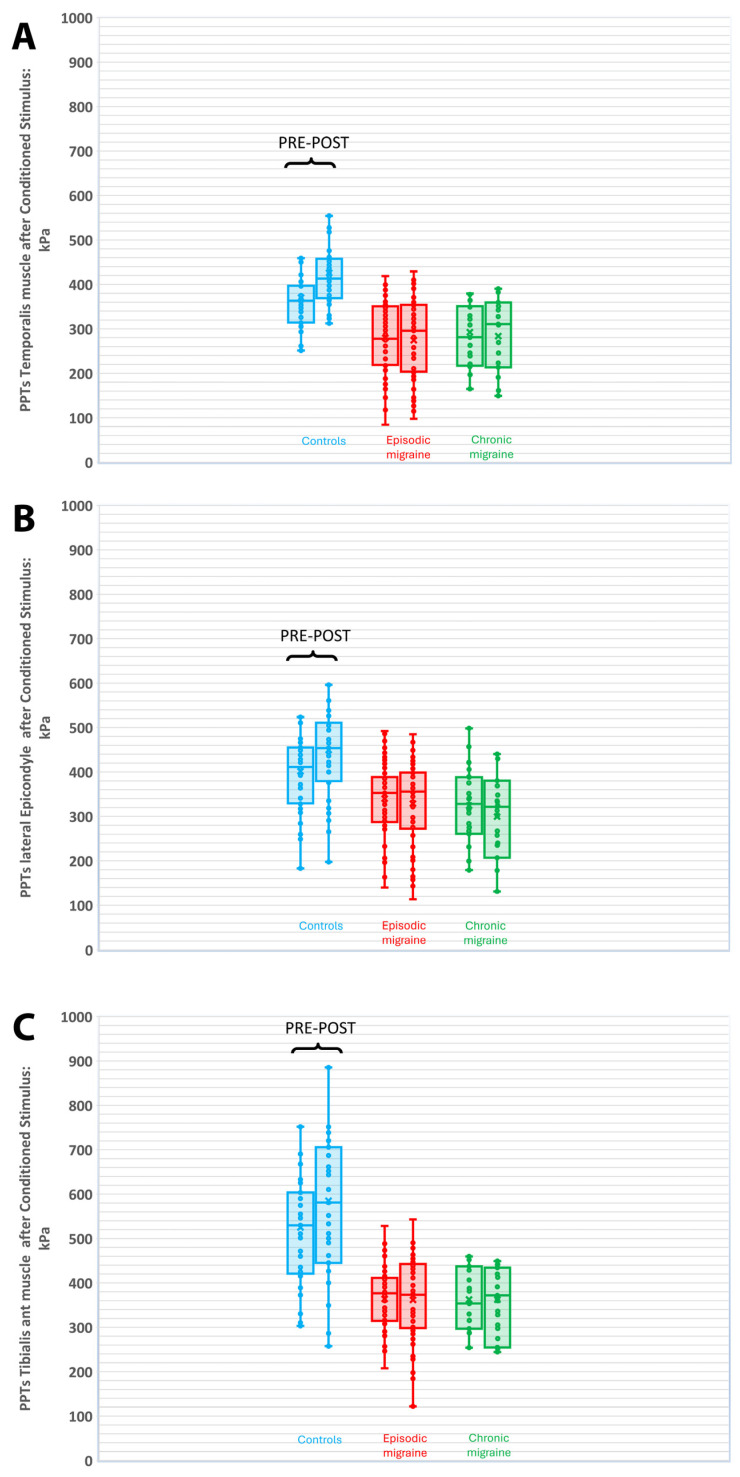
Changes in pressure pain thresholds (PPTs) in the temporalis muscle (**A**), lateral epicondyle (**B**), and tibialis anterior muscle (**C**) before and after the conditioned stimulus (cold-water immersion) in women with episodic migraine, women with chronic migraine, and healthy controls.

A significant group × time interaction was found (Wilk’s λ = 0.588, F[2,199] = 69.756, *p* < 0.001, n^2^p = 0.412, 1 − β = 0.999) without a significant effect of the following covariates: educational level (Wilk’s λ = 0.980, F[1,199] = 4.144, *p* = 0.053, n^2^p = 0.020, 1 − β = 0.526), employment status (Wilk’s λ = 0.997, F[1,199] = 0.652, *p* = 0.420, n^2^p = 0.003, 1 − β = 0.127), DSS (Wilk’s λ = 1.000, F[1,199] = 0.055, *p* = 0.815, n^2^p = 0.000, 1 − β = 0.056), ROCF_Recall (Wilk’s λ = 0.996, F[1,199] = 0.835, *p* = 0.362, n^2^p = 0.004, 1 − β = 0.149), ROCF_TimeCopy (Wilk’s λ = 1.000, F[1,199] = 0.087, *p* = 0.769, n^2^p = 0.000, 1 − β = 0.060), Decoding_FDT (Wilk’s λ = 0.980, F[1,199] = 3.986, *p* = 0.057, n^2^p = 0.020, 1 − β = 0.511), Retrieving_FDT (Wilk’s λ = 1.000, F[1,199] = 0.071, *p* = 0.789, n^2^p = 0.000, 1 − β = 0.058), and Zoo Map test (Wilk’s λ = 0.986, F[1,199] = 2.739, *p* = 0.099, n^2^p = 0.014, 1 − β = 0.377). PPTs were higher after the conditioned stimulus (mean difference: 59.7 kPa, 95%CI 50.7 to 68.8, *p* < 0.001) in control women, whereas they were lower after the conditioned stimulus in women with chronic migraine (mean difference: 13.4 kPa, 95%CI 4.2 to 22.8, *p* = 0.005) in comparison to PPTs before the stimulus (Table 3). No significant changes in PPTs before/after the conditioned stimulus were observed in women with episodic or chronic migraine (Figure 6A). Thus, the mechanical CPM paradigm within the trigeminal area revealed an increase of 16.2% (0.1%) in control women and a decrease of −3.0% (0.15%) in the CPM activation index in women with chronic migraine (*p* < 0.001).

### 3.3. Mechanical CPM in Extra-Trigeminal Area (PPTs Lateral Epicondyle)

The repeated-measures MANCOVA (the estimated marginal means and standard deviations can be seen in Table 4) showed a significant time effect for PPTs (Wilk’s λ= 0.963, F[1,200] = 7.760, *p* = 0.006, n^2^p = 0.037, 1 − β = 0.792). However, post hoc analyses revealed no significant differences in PPTs before/after the conditioned stimulus (mean difference: 4.1 kPa, 95%CI −2.0 to 10.2, *p* = 0.188).

A significant group × time interaction (Wilk’s λ = 0.674, F[2,200] = 48.331, *p* < 0.001, n^2^p= 0.326, 1 − β = 0.999) was found without a significant effect of the following covariates: educational level (Wilk’s λ = 0.979, F[1,199] = 4.288, *p* = 0.056, n^2^p = 0.021, 1 − β = 0.540), employment status (Wilk’s λ = 0.996, F[1,199] = 0.868, *p* = 0.353, n^2^p = 0.004, 1 − β = 0.153), DSS (Wilk’s λ = 0.987, F[1,199] = 2.602, *p* = 0.108, n^2^p = 0.013, 1 − β = 0.362), ROCF_Recall (Wilk’s λ = 0.993, F[1,199] = 1.322, *p* = 0.252, n^2^p = 0.007, 1 − β = 0.208), ROCF_TimeCopy (Wilk’s λ = 1.000, F[1,199] = 0.009, *p* = 0.926, n^2^p = 0.000, 1 − β = 0.051), Decoding_FDT (Wilk’s λ = 0.954, F[1,199] = 1.500, *p* = 0.389, n^2^p = 0.006, 1 − β = 0.145), Retrieving_FDT (Wilk’s λ= 0.988, F[1,199] = 2.369, *p* = 0.125, n^2^p = 0.012, 1 − β = 0.334), and Zoo Map test (Wilk’s λ = 1.000, F[1,199] = 0.003, *p* = 0.957, n^2^p = 0.000, 1 − β = 0.050). PPTs were higher after the conditioned stimulus (mean difference: 49.4 kPa, 95%CI 38.5 to 60.5, *p* < 0.001) than before the stimulus in control women, but they were lower after the conditioned stimulus in women with episodic (mean difference: −12.5 kPa, 95%CI −23.3 to −1.8, *p* = 0.022) or with chronic (mean difference: −24.6 kPa, 95%CI −35.7 to −13.5, *p* < 0.001) migraine (Table 4, Figure 6B).

The mechanical CPM paradigm within the extra-trigeminal area revealed an increase of 12.3% (0.15%) in the CPM activation index in control women and a decrease of −3.8% (0.15%) and of −7.6% (0.15%) in women with episodic or chronic migraine, respectively (*p* < 0.001).

### 3.4. Mechanical CPM in Distant Area (PPTs Tibialis Anterior Muscle)

The repeated-measures MANCOVA analyses (the estimated marginal means and standard deviations are depicted in Table 5) revealed a significant time effect for PPTs (Wilk’s λ = 0.974, F[1,200] = 5.240, *p* = 0.023, n^2^p = 0.026, 1 − β = 0.625): PPTs were higher after the conditioned stimulus (mean difference: 19.3 kPa, 95%CI 11.8 to 26.8, *p* < 0.001) than before.

A significant group × time interaction (Wilk’s λ = 0.751, F[2,200] = 33.110, *p* < 0.001, n^2^p = 0.249, 1 − β = 0.999) was found without a significant effect of the following covariates: educational level (Wilk’s λ = 0.992, F[1,199] = 1.624, *p* = 0.204, n^2^p = 0.008, 1 − β = 0.245), employment status (Wilk’s λ = 0.966, F[1,199] = 1.963, *p* = 0.353, n^2^p = 0.504, 1 − β = 0.257), DSS (Wilk’s λ = 0.999, F[1,199] = 0.294, *p* = 0.588, n^2^p = 0.001, 1 − β = 0.084), ROCF_Recall (Wilk’s λ = 0.981, F[1,199] = 3.916, *p* = 0.053, n^2^p = 0.019, 1 − β = 0.504), ROCF_ TimeCopy (Wilk’s λ = 0.963, F[1,199] = 1.742, *p* = 0.566, n^2^p = 0.002, 1 − β = 0.191), Decoding_FDT (Wilk’s λ = 0.998, F[1,199] = 0.444, *p* = 0.506, n^2^p = 0.002, 1 − β = 0.102), Retrieving_FDT (Wilk’s λ= 0.994, F[1,199] = 1.296, *p* = 0.256, n^2^p = 0.006, 1 − β = 0.205), and Zoo Map test (Wilk’s λ = 0.992, F[1,199] = 1.668, *p* = 0.198, n^2^p = 0.008, 1 − β = 0.251). Pairwise comparisons showed that PPT values increased after the conditioned stimulus in control women (mean difference: 65.3 kPa, 95%CI 51.9 to 78.75, *p* < 0.001) but without significant changes (with a tendency to a decrease) in women with episodic (mean difference: −3.3 kPa) or chronic (mean difference: −3.9 kPa) migraine (Table 5, Figure 6C). The mechanical CPM paradigm in distant pain-free area revealed an increase of 10.7% (0.15%) in the CPM activation index in control women and a decrease of −0.5% (0.1%) in women with chronic migraine and −0.3% (0.1%) in women with episodic migraine, respectively (*p* < 0.001).

### 3.5. Thermal CPM in the Trigeminal Area (CPT and HPT Frontalis Muscle)

The repeated-measures MANCOVA analyses did not find a significant time effect for CPT (Wilk’s λ = 0.984, F[1,200] = 3.183, *p* = 0.076, n^2^p = 0.016, 1 − β = 0.427, Table 6) or HPT (Wilk’s λ = 0.998, F[1,200] = 0.362, *p* = 0.548, n^2^p = 0.002, 1 − β = 0.092, Table 7).

**Figure 7:**
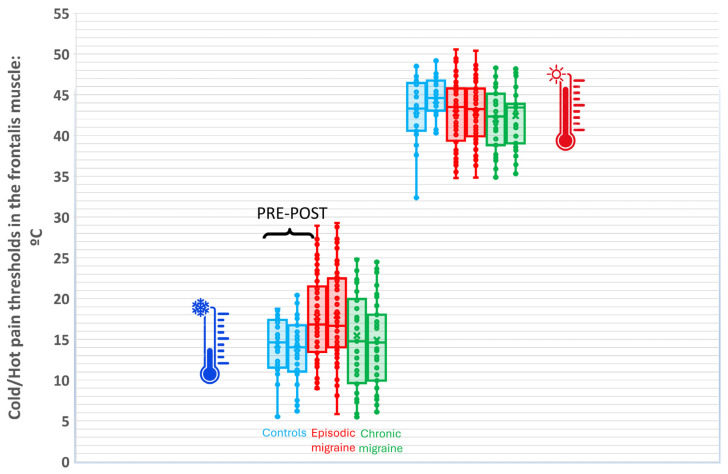
Changes in cold pain thresholds (down of the figure) and hot pain thresholds (up of the figure) in the frontalis area before and after the conditioned stimulus (cold-water immersion) in women with episodic migraine, women with chronic migraine, and healthy controls.

A significant group × time interaction was found for CPT (Wilk’s λ = 0.971, F[2,201] = 3.043, *p* = 0.048, n^2^p = 0.029, 1 − β = 0.584) without a significant effect of the following covariates: educational level (Wilk’s λ = 0.972, F[1,199] = 2.814, *p* = 0.104, n^2^p = 0.018, 1 − β = 0.570), employment status (Wilk’s λ = 0.980, F[1,199] = 2.032, *p* = 0.353, n^2^p = 0.020, 1 − β = 0.317), DSS (Wilk’s λ = 0.997, F[1,199] = 0.515, *p* = 0.474, n^2^p = 0.003, 1 − β = 0.110), ROCF_Recall (Wilk’s λ = 0.975, F[1,199] = 3.016, *p* = 0.183, n^2^p = 0.014, 1 − β = 0.406), ROCF_ TimeCopy (Wilk’s λ = 0.994, F[1,199] = 1.127, *p* = 0.290, n^2^p = 0.006, 1 − β = 0.184), Decoding_FDT (Wilk’s λ = 0.990, F[1,199] = 2.102, *p* = 0.149, n^2^p = 0.010, 1 − β = 0.303), Retrieving_FDT (Wilk’s λ= 0.999, F[1,199] = 0.214, *p* = 0.644, n^2^p = 0.001, 1 − β = 0.075), and Zoo Map test (Wilk’s λ = 0.987, F[1,199] = 2.544, *p* = 0.112, n^2^p = 0.013, 1 − β = 0.354). Similarly, a significant group * time interaction was also found for HPT (Wilk’s λ = 0.955, F[2,201] = 4.788, *p* = 0.009, n^2^p = 0.045, 1 − β = 0.791) without a significant effect of the following covariates: educational level (Wilk’s λ = 0.963, F[1,199] = 2.612, *p* = 0.201, n^2^p = 0.007, 1 − β = 0.284), employment status (Wilk’s λ = 0.992, F[1,199] = 1.572, *p* = 0.211, n^2^p = 0.008, 1 − β = 0.239), DSS (Wilk’s λ = 1.000, F[1,199] = 0.027, *p* = 0.869, n^2^p = 0.000, 1 − β = 0.053), ROCF_Recall (Wilk’s λ = 0.987, F[1,199] = 2.568, *p* = 0.111, n^2^p = 0.013, 1 − β = 0.358), ROCF_ TimeCopy (Wilk’s λ = 0.993, F[1,199] = 1.432, *p* = 0.233, n^2^p = 0.007, 1 − β = 0.222), Decoding_FDT (Wilk’s λ = 1.000, F[1,199] = 0.022, *p* = 0.883, n^2^p = 0.000, 1 − β = 0.052), Retrieving_FDT (Wilk’s λ= 1.000, F[1,199] = 0.008, *p* = 0.928, n^2^p = 0.000, 1 − β = 0.051), and Zoo Map test (Wilk’s λ = 0.999, F[1,199] = 0.292, *p* = 0.589, n^2^p = 0.001, 1 − β = 0.084). Post hoc analyses revealed that CPTs decreased in a non-significant way in all groups (Table 6, Figure 7). Thus, pairwise post hoc comparison showed that HPTs were higher (mean difference: 1.3 °C, 95%CI 0.95 to 1.8, *p* < 0.001) after the conditioned stimulus in control women (Table 7), but no significant changes were seen in women with episodic (mean difference −0.5 °C) or chronic migraine (mean difference −0.35 °C, Figure 7).

The thermal CPM paradigm in the trigeminal area revealed an increase of 3.3% (0.5%) in the heat CPM activation index in control women and 1.1% (0.3%) in women with chronic migraine but without a significant change in women with episodic migraine (*p* < 0.001).

## 4. Discussion

Women with migraine, either episodic or chronic, showed CPM deficits particularly with pain thresholds as compared to control women. Thus, neurocognitive performance on different domains such as attention levels and/or executive functions did not influence CPM activity.

### 4.1. Impaired Conditioned Pain Modulation in Migraine

The current study revealed CPM deficits in sensitivity to pressure pain but not to thermal pain in women with migraine as compared to healthy women. Healthy women exhibited an increase of approximately 10% in the CPM activation index across all tested sites, reflecting effective descending inhibitory control; however, women with migraine displayed either no modulation or even negative changes in the CPM activation index.

The literature regarding CPM in migraine remains inconsistent. Systematic reviews generally concluded that CPM efficiency is comparable between patients with migraine and controls [9,10]. Inconsistencies across studies have been attributed to methodological issues such as the following: 1, small samples (often 8–30 per group) and combining both sexes; 2, lack of differentiation between episodic/chronic migraine; and 3, use of several diverse CPM paradigms [9,10].

First, small sample sizes in previous studies may have resulted in Type II errors, reducing the ability to detect true differences. Further, previous studies pooled men and women, despite known sex-related differences in migraine prevalence and nociceptive processing [30]. In fact, some authors suggested that men exhibit stronger CPM responses than women, whereas others reported no differences [31]. Martel et al. highlighted that CPM paradigms yield reliable results in women but not in men, suggesting sex-mixing samples may have contributed to variability in outcomes [32].

Second, earlier investigations often did not distinguish between episodic and chronic migraine. Some authors hypothesize that chronic migraine can involve more pronounced central sensitization mechanisms. Our findings, however, showed no CPM differences between episodic and chronic migraine, suggesting that descending pain inhibition is similarly impaired in both forms of migraine. The meta-analysis conducted by van Welie et al. found that available data about CPM efficiency in chronic migraine is limited due to the small number of studies and small samples used in previous studies [10]. The lack of differences in sensitization between women with episodic and chronic migraine agrees with a meta-analysis showing no significant differences between both migraine forms in widespread pressure pain hypersensitivity, a clinical manifestation of pain facilitation [7]. Current results would support the “headache continuum” hypothesis whereby both forms of migraine could share similar patterns of altered nociceptive processing [33].

Third, stimulus and anatomical site dependency are likely to contribute to heterogeneity in CPM response. Most studies used thermal stimuli, two used electrical stimuli, and one used a mechanical stimulus, all applied on the trigeminal area [9,10]. Thus, CPM paradigms used in previous studies were different: laser-evoked potential in all studies using thermal stimuli, hand–water immersion in both studies using electrical stimuli and cuff-occlusion paradigm in the study using mechanical stimuli [9,10]. Our study is the first one to explore CPM simultaneously with pressure and thermal modalities across trigeminal, extra-trigeminal, and remote pain-free sites, using a paradigm with established reliability [26]. Interestingly, we observed that the application of mechanical stimulus was able to identify an impaired CPM in women with episodic/chronic migraine, whereas the application of thermal stimuli (heat or cold) did not. This finding is consistent with the meta-analysis by Nahman-Averbuch et al. showing high heterogeneity in pain perception between individuals with migraine and healthy controls depending on the stimuli applied [6]. For instance, patients with migraines exhibit higher pain sensitivity to pressure but not cold or electrical pain hypersensitivity [6]. These authors suggested that a different response depending on the sensory modality may be related to different activation at the level of peripheral receptors/nerve fibers (i.e., mechanical simulation is mainly mediated by C-fiber pathway, whereas thermal stimulation is mainly mediated by Aδ fiber pathway) or even central pathways [6]. Thus, the results of our study suggest that the mechanical stimulus is more sensitive for evaluating CPM activity than the thermal stimulus in patients with migraine.

### 4.2. Neurocognitive Processes/Executive Functions and Conditioned Pain Modulation

This is the first study to examine how cognitive processes and executive functions influence CPM in individuals with migraine. Our findings indicated that neither cognitive performance nor executive functions modulate CPM response in women with migraine.

We observed deficits in performance in some but not all neurocognitive processes and executive functions in women with episodic or chronic migraine when compared with healthy women. For example, we found deficits in visual perception, mental inhibition, and planning and decision-making but not in selective attention between migraine women and healthy controls. These results are overall consistent with the current literature as most meta-analyses have concluded that deficits in cognitive performance and executive functions in people with migraine are domain-specific. For instance, Kaiser Pinotti et al. found worse performance in domains such as attention, working memory, or mental flexibility, whereas other domains such as verbal fluency, inhibitory control, or response maintenance showed comparable performance between patients with migraine and controls [13]. Similarly, Pizer et al. observed worse performance in processing speed, visuospatial/construction, simple and complex attention, learning/memory, and language but similar performance in orientation, motor, and intelligence between patients with migraine and controls [12].

A growing body of evidence suggests that executive functions and descending pain inhibition are interconnected, likely due to overlapping neural networks. In particular, the anterior cingulate cortex (ACC) is a key brain area involved in descending pain inhibition and cognitive/emotional regulation [34,35,36]. Rischer et al. reported that better performance in executive functions was associated with higher activation of prefrontal brainstem areas during a distraction task, suggesting that preservation of executive functions may enhance descending pain inhibition associated with more effective pain distraction [37]. Similarly, Lithfous et al. found that old adults with higher executive functioning displayed more efficient endogenous pain modulation than those with poorer executive test outcomes [38]. Our study is the first to assess the relationship between neurocognitive abilities, executive functioning, and CPM function in people with migraine. Current results show that lower performance in some neurocognitive processes or executive functions did not alter CPM functioning in women with episodic or chronic migraine. One possible explanation could be that the heterogeneous changes in brain structure and functional connectivity observed in patients with migraine lead to potential compensatory neural mechanisms in descending pain inhibition control [39]. Thus, studies investigating the association of neurocognitive evaluations with functional changes in the brain could elucidate this hypothesis. Another hypothesis would be that association of neurocognitive processes and executive functions with CPM could be paradigm-dependent, since women with migraine exhibited impaired pain inhibition to mechanical but not thermal stimuli. To date, no study has tested this idea. Thus, current results suggest that CPM impairment observed in women with migraine is likely related to specific pain processing pathways rather than general neurocognitive function.

### 4.3. Strengths and Limitations of the Study

This current case–control study has several strengths, including an adequate sample size, the distinction between episodic or chronic migraine, and the control of executive functions and neurocognitive processes in the analysis. Nevertheless, the cross-sectional nature of the study prevents drawing conclusions regarding a potential causal direction of the findings. Second, current results should not be extrapolated to men with migraine, as sex-related differences in pain modulation, mostly associated with hormonal influence, are documented. For instance, an increased mechanical pain sensitivity (i.e., lower PPTs) has been found to be higher during menstruation [40]. Although we did not collect data on the menstrual cycle phase in our study, it has been previously found that differences in CPM between migraineurs and healthy volunteers did not vary over the menstrual cycle [41]. Third, we did not collect data on mood disorders, e.g., anxiety or depressive levels, which could have a potential association with neurocognitive variables or altered nociceptive processing. Nevertheless, current evidence does not support an association between mood disorders and CPM in either healthy subjects [42] or people with spinal chronic pain [43]. Fourth, medication intake can have an effect on the assessment of pain processing outcomes; nevertheless, it should be noted that no change in medication intake was conducted. Thus, no differences in either prophylactic or symptomatic (abortive) medication consumption was seen between women with episodic or chronic migraine. Finally, it should be considered that the subjective experience of pain and its impact can be different among patients with the same pain condition, e.g., migraine. It has been found that individuals with chronic headaches perceive their pain as a behavior drive leading to a strain of their relationships [44]. It is possible that a worse subjective experience of pain can enhance pain inhibition by descending mechanisms; however, we did not collect any subjective measurement of the pain experience in our sample of women with migraine.

## 5. Conclusions

This case–control study found that women with episodic or chronic migraine exhibited impaired CPM against mechanical but not thermal stimuli as compared with control women. Thus, neurocognitive (e.g., attention) processes or executive functions (e.g., working memory, mental inhibition) did not modulate CPM activity in women with episodic or chronic migraine. The results from this study suggest that neurocognitive processes/executive functions do not modulate descending pain modulation in women with migraine. Thus, clinical trials investigating if treatment of neurocognitive deficits can help to modulate altered nociceptive processing could help to elucidate the relevance of the current findings.

## Figures and Tables

**Table 1:** Sociodemographic and data of the sample.

	Chronic Migraine (*n* = 70)	Episodic Migraine (*n* = 70)	Controls (*n* = 70)		
Sociodemographic Data					
	*n* (%)	*n* (%)	*n* (%)	χ^2^	*p*
Educational level					
Primary	0 (0%)	4 (5.7%)	4 (5.7%)	14.577	0.006
Secondary	31 (44.3%)	16 (22.9%)	14 (20%)		
Higher education	39 (55.7%)	50 (71.4%)	52 (74.30%)		
Employment status					
Student	6 (8.6%)	3 (4.3%)	0 (0%)	24.400	<0.001
Working	52 (74.3%)	56 (80%)	54 (77.1%)		
Unemployed	12 (17.1%)	7 (10%)	4 (5.7%)		
Retired	0 (0%)	4 (5.7%)	12 (17.1%)		
Marital status					
Single	25 (35.7%)	26 (37.1%)	20 (28.6%)		
Married	33 (47.1%)	36 (51.4%)	36 (51.4%)	4.545	0.603
Divorced	6 (8.6%)	2 (2.9%)	8 (11.4%)		
Widowed	6 (8.6%)	6 (8.6%)	6 (8.6%)		
**Clinical Data**					
	**Mean (SD)**	**Mean (SD)**	**Mean (SD)**	**F**	***p***
Age (years)	46.1 (5.2)	48.1 (7.2)	49.1 (9.6)	2.905	0.057
Intensity (NPRS, 0–10)	8.6 (1.4)	7.6 (1.55)	---		
Frequency (days/month)	16.5 (6.4)	3.5 (2.7)	---		
Duration (hours/attack)	33.1 (20.3)	25.3 (23.9)	---		
	***n* (%)**	***n* (%)**	***n* (%)**	**χ^2^**	***p***
Side of migraine				1.602	0.445
Left	12 (17%)	14 (20%)	---		
Right	19 (27%)	17 (24%)	---		
Bilateral	39 (56%)	39 (56%)	---		
Preventive Treatment				0.560	0.454
Yes (Amitriptyline)	52 (74.3%)	48 (68.6%)	---		
No	18 (25.7%)	22 (31.4%)	---		
Symptomatic Treatment				0.548	0.459
Yes	59 (84.3%)	62 (88.6%)	---		
No	11 (15.7%)	8 (11.4%)	---		
Type of Symptomatic Treatment				0.180	0.672
Painkillers	5 (8.5%)	4 (6.5%)	---		
NSAIDs	54 (91.5%)	58 (93.5%)	---		

*n*: number of subjects, SD: standard deviation; NSAIDs: Non-steroidal anti-inflammatory drugs.

**Table 2:** Neurocognitive performance and executive functions of the sample.

	Chronic Migraine (*n* = 70)	Episodic Migraine (*n* = 70)	Controls (*n* = 70)		
	Mean (SD)	Mean (SD)	Mean (SD)	F	*p*
d2_TR	432.7 (8.7)	423.4 (11.6)	411.3 (8.9)	0.871	0.420
d2_TA	148.45 (4.5)	156.2 (5.3)	144.8 (4.2)	0.457	0.634
d2_O	32.4 (3.6)	44.5 (4.9)	28.5 (3.8)	0.896	0.410
d2_C	2.3 (5.5)	19.05 (11.2)	3.9 (8.7)	1.257	0.287
d2_TOT	398.0 (8.1)	388.4 (9.9)	378.8 (8.8)	0.668	0.514
d2_CON	146.2 (4.8)	150.2 (4.3)	139.8 (4.4)	0.339	0.713
d2_VAR	13.4 (5.6)	29.8 (6.7)	14.7 (7.6)	1.251	0.288
DSF	7.9 (1.6)	8.1 (2.0)	8.2 (2.2)	0.344	0.709
DSB	7.5 (1.4)	7.0 (1.8)	7.7 (2.2)	2.564	0.079
DSS #	7.6 (1.8)	8.6 (2.2)	8.2 (2.3)	3.431	0.034
ROCF_Copy	30.5 (4.2)	28.6 (5.8)	29.4 (5.8)	2.263	0.107
ROCF_Recall #	12.7 (5.1)	14.6 (6.1)	15.6 (7.3)	3.985	0.020
ROCF_TimeCopy #	2.4 (1.6)	2.1 (0.7)	1.9 (0.6)	3.630	0.028
Symbol Search	32.0 (8.2)	34.2 (9.3)	32.7 (10.2)	1.070	0.345
Decoding_FDT #	20.5 (4.2)	18.65 (4.2)	17.9 (3.9)	7.565	0.001
Retrieving_FDT #	24.5 (9.1)	21.2 (3.9)	20.8 (4.0)	7.548	0.001
Inhibiting_FDT	34.8 (12.7)	32.3 (7.3)	33.3 (9.02)	1.124	0.327
Shifting_FDT	42.7 (11.2)	43.5 (12.9)	41.5 (11.0)	0.494	0.611
Zoo Map test #	13.4 (3.3)	12.4 (3.15)	11.5 (3.0)	6.056	0.003

SD: standard deviation; d2_TR: total number of items answered; d2_TA: number of items answered correctly; d2_O: errors of omission committed; d2_C: commission errors made; d2_TOT: number of elements processed minus the total number of errors committed; d2_CON: number of relevant elements marked minus the number of commissions; d2_VAR: variation index d2; DSF: digit span forward; DSB: digit span backward; DSS: digit span sequencing; ROCF_Copy: direct scoring in the copy phase of the Rey–Osterrieth Complex Figure; ROCF_Recall: direct scoring in the delayed Recall phase of the Rey–Osterrieth Complex Figure; Symbol Search: direct scoring of correctly answered items; Decoding_FDT: time in seconds to read all numeric items; Retrieving_FDT: time in seconds to read all non-numeric items; Inhibiting_FDT: time in seconds to read numeric items; Shifting_FDT: time in seconds to read non-numeric items; Zoo Map Test: direct score in carrying out the planning test. # Significant differences among groups.

**Table 3:** Pressure pain thresholds (PPTs, kPa) before and after the conditioned stimulus (cold pressor test) in the temporalis muscle.

	Chronic Migraine (*n* = 70)	Episodic Migraine (*n* = 70)	Controls (*n* = 70)	Univariate Tests			
Intergroup Differences							
PPT Values	Mean (SD)	Mean (SD)	Mean (SD)	F	*p*	η^2^p	1 − β
Before	276.9 (9.6)	282.0 (8.9)	383.2 (9.4)	38.906	<0.001	0.281	0.999
After	263.4 (10.0)	277.9 (9.3)	442.9 (9.8)	98.135	<0.001	0.497	0.999
**Intragroup Differences**							
	**Before Stimulus**	**After Stimulus**					
**Group**	**Mean (SD)**	**Mean (SD)**		**F**	***p***	**η^2^p**	**1 − β**
Chronic migraine	276.9 (9.6)	263.2 (10.0)		8.139	0.005	0.039	0.810
Episodic migraine	282.0 (8.9)	277.9 (9.3)		0.872	0.352	0.004	0.153
Control subjects	383.2 (9.4)	442.9 (9.8)		168.713	<0.001	0.459	0.999

**Table 4:** Pressure pain thresholds (PPTs, kPa) before and after the conditioned stimulus (cold pressor test) in the lateral epicondyle.

	Chronic Migraine (*n* = 70)	Episodic Migraine (*n* = 70)	Controls (*n* = 70)	Univariate Tests			
Intergroup Differences							
PPT Values	Mean (SD)	Mean (SD)	Mean (SD)	F	*p*	η^2^p	1 − β
Before	313.9 (11.3)	337.9 (10.5)	413.6 (11.0)	20.163	<0.001	0.168	0.999
After	289.2 (12.7)	325.5 (11.8)	463.2 (12.4)	49.801	<0.001	0.334	0.999
**Intragroup Differences**							
	**Before Stimulus**	**After Stimulus**					
**Group**	**Mean (SD)**	**Mean (SD)**		**F**	***p***	**η^2^p**	**1 − β**
Chronic migraine	313.9 (11.3)	289.2 (12.7)		17.480	<0.001	0.081	0.986
Episodic migraine	337.9 (10.5)	325.5 (11.8)		5.225	0.023	0.026	0.624
Control subjects	413.6 (11.0)	463.2 (12.4)		74.031	<0.001	0.271	0.999

**Table 5:** Pressure pain thresholds (PPTs, kPa) before and after the conditioned stimulus (cold pressor test) in the tibialis anterior muscle.

	Chronic Migraine (*n* = 70)	Episodic Migraine (*n* = 70)	Controls (*n* = 70)	Univariate Tests			
Intergroup Differences							
PPT Values	Mean (SD)	Mean (SD)	Mean (SD)	F	*p*	η^2^p	1 − β
Before	356.1 (13.2)	363.6 (12.2)	531.5 (12.8)	56.705	<0.001	0.363	0.999
After	349.4 (15.9)	360.9 (14.8)	599.0 (15.5)	78.079	<0.001	0.440	0.999
**Intragroup Differences**							
	**Before Stimulus**	**After Stimulus**					
**Group**	**Mean (SD)**	**Mean (SD)**		**F**	***p***	**η^2^p**	**1 − β**
Chronic migraine	356.1 (13.2)	349.4 (15.9)		0.869	0.352	0.004	0.153
Episodic migraine	363.6 (12.2)	360.9 (14.8)		0.171	0.679	0.001	0.070
Control subjects	531.5 (12.8)	599.0 (15.5)		92.926	<0.001	0.318	0.999

**Table 6:** Cold pain thresholds (CPT, °C) before and after the conditioned stimulus (cold pressor test) in the frontalis muscle.

	Chronic Migraine (*n* = 70)	Episodic Migraine (*n* = 70)	Controls (*n* = 70)	Univariate Tests			
Intergroup Differences							
CPTs Values	Mean (SD)	Mean (SD)	Mean (SD)	F	*p*	η^2^p	1 − β
Before	15.0 (0.6)	18.1 (0.55)	14.5 (0.6)	11.527	<0.001	0.104	0.993
After	14.8 (0.65)	18.1 (0.6)	13.8 (0.6)	13.980	<0.001	0.123	0.998
**Intragroup Differences**							
	**Before Stimulus**	**After Stimulus**					
**Group**	**Mean (SD)**	**Mean (SD)**		**F**	***p***	**η^2^p**	**1 − β**
Chronic migraine	15.0 (0.6)	14.8 (0.65)		0.111	0.739	0.001	0.063
Episodic migraine	18.1 (0.55)	18.1 (0.6)		0.011	0.915	0.000	0.051
Control subjects	14.51 (0.6)	13.8 (0.6)		3.024	0.084	0.015	0.409

**Table 7:** Heat pain thresholds (HPT, °C) before and after the conditioned stimulus (cold pressor test) in the frontalis muscle.

	Chronic migraine (*n* = 70)	Episodic migraine (*n* = 70)	Controls (*n* = 70)	Univariate tests			
Intergroup Differences							
HPTs Values	Mean (SD)	Mean (SD)	Mean (SD)	F	*p*	η^2^p	1 − β
Before	42.3 (0.5)	42.5 (0.45)	43.0 (0.5)	0.451	0.637	0.005	0.123
After	42.7 (0.4)	42.6 (0.4)	44.3 (0.4)	5.473	0.005	0.052	0.845
**Intragroup Differences**							
	**Before Stimulus**	**After Stimulus**					
**Group**	**Mean (SD)**	**Mean (SD)**		**F**	***p***	**η^2^p**	**1 − β**
Chronic migraine	42.3 (0.5)	42.7 (0.4)		2.791	0.096	0.014	0.383
Episodic migraine	42.5 (0.45)	42.6 (0.4)		0.033	0.855	0.000	0.054
Control subjects	43.0 (0.5)	44.3 (0.4)		36.726	<0.001	0.156	0.999

## Data Availability

Materials and analysis code for this study are not available in any repository; however, we will make our data accessible upon request to the corresponding author.
